# Neural substrates underlying reconcentration for the preparation of an appropriate cognitive state to prevent future mistakes: a functional magnetic resonance imaging study

**DOI:** 10.3389/fnhum.2015.00603

**Published:** 2015-11-03

**Authors:** Naoki Miura, Takayuki Nozawa, Makoto Takahashi, Ryoichi Yokoyama, Yukako Sasaki, Kohei Sakaki, Ryuta Kawashima

**Affiliations:** ^1^Department of Information and Communication Engineering, Faculty of Engineering, Tohoku Institute of TechnologySendai, Japan; ^2^Department of Ubiquitous Sensing, Institute of Development, Aging and Cancer (IDAC), Tohoku UniversitySendai, Japan; ^3^Department of Management Science and Technology, Graduate School of Engineering, Tohoku UniversitySendai, Japan; ^4^Department of Functional Brain Imaging, Institute of Development, Aging and Cancer (IDAC), Tohoku UniversitySendai, Japan; ^5^Faculty of Medicine, Kobe University School of MedicineKobe, Japan

**Keywords:** functional MRI, cognitive control, reconcentration, post-error behavioral adjustment, medial frontal cortex

## Abstract

The ability to reconcentrate on the present situation by recognizing one’s own recent errors is a cognitive mechanism that is crucial for safe and appropriate behavior in a particular situation. However, an individual may not be able to adequately perform a subsequent task even if he/she recognize his/her own error; thus, it is hypothesized that the neural mechanisms underlying the reconcentration process are different from the neural substrates supporting error recognition. The present study performed a functional magnetic resonance imaging (fMRI) analysis to explore the neural substrates associated with reconcentration related to achieving an appropriate cognitive state, and to dissociate these brain regions from the neural substrates involved in recognizing one’s own mistake. This study included 44 healthy volunteers who completed an experimental procedure that was based on the Eriksen flanker task and included feedback regarding the results of the current trial. The hemodynamic response induced by each instance of feedback was modeled using a combination of the successes and failures of the current and subsequent trials in order to identify the neural substrates underlying the ability to reconcentrate for the next situation and to dissociate them from those involved in recognizing current errors. The fMRI findings revealed significant and specific activation in the dorsal aspect of the medial prefrontal cortex (MFC) when participants successfully reconcentrated on the task after recognizing their own error based on feedback. Additionally, this specific activation was clearly dissociated from the activation foci that occurred during error recognition. These findings indicate that the dorsal aspect of the MFC may be a distinct functional region that specifically supports the reconcentration process and that is associated with the prevention of successive errors when a human subject recognizes his/her own mistake. Furthermore, it is likely that this reconcentration mechanism acts as a trigger to perform successful post-error behavioral adjustments.

## Introduction

To err is human. However, humans can learn from their own mistakes. For example, when people recognize their own behavioral error, they can reconcentrate and improve their own cognitive state so that the error will not be repeated during subsequent performance. This cognitive mechanism has been referred to as a type of “cognitive set” (Sakai, 2008) or “cognitive readiness” (Morrison and Fletcher, 2002) and it is expected that reconcentration mechanism is a preparatory process to make a correct response after recognizing his/her own error, therefore, this mechanism would work as a trigger to drive a post-error behavioral adjustment (Danielmeier and Ullsperger, 2011). Even though human errors are inevitable, when this cognitive mechanism is appropriately activated, humans can avoid serious accidents that may occur due to a chain of bad incidents. Thus, it is expected that a better understanding of this mechanism will not only contribute to the elucidation of human cognition by fostering an awareness of one’s own cognitive state, but that it will also aid in the development of human factors research.

It is plausible that the cognitive mechanisms required to reconcentrate on the present situation and those needed to recognize one’s own error differ, because humans do not always prepare an appropriate cognitive state after an error and sometimes repeatedly make the same kinds of errors. Similarly, it may also be expected that humans can avoid repeating previous errors due to the achievement of a cognitive state that is likely driven by their ability to recognize a mistake. Ridderinkhof et al. (2004) suggested that the medial prefrontal cortex (MFC) is an important brain region involved in the recognition of response errors and negative feedback and furthermore, Ullsperger et al. (2010) found that the anterior insular cortex is also crucial for error perception. Additionally, the occurrence of a response error has been shown to be represented by a decrease in cortical deactivation in default mode regions in conjunction with reduced cortical activation in task-related regions (Weissman et al., 2006; Eichele et al., 2008; King et al., 2010).

The role of the MFC during cognitive tasks is also thought to pertain to the adjustment of performance after recognizing behavioral errors (Ridderinkhof et al., 2004), as evidenced by the post-error slowing effect (Danielmeier and Ullsperger, 2011; Ullsperger et al., 2014). Hester et al. (2007) found that the activation of the bilateral prefrontal cortices and the posterior MFC was associated with post-error slowing during the periods following error trials. Additionally, Li et al. (2008) observed a relationship between activity in the ventrolateral prefrontal cortex and the slowing of reaction time (RT) after errors. Hendrick et al. (2010) suggested an existence of distinct pathways for post-error and post-non-error conflict processing in cognitive control. Danielmeier et al. (2011) also reported a functional interaction between the posterior MFC and task-related cortical regions during the processing of post-error adaptations. However, because a majority of these studies focused on changes in cortical activity subsequent to the occurrence of errors, it remains unclear whether specific types of cognitive control are necessary for reconcentration in order to prepare for post-error adaptation after recognizing one’s own error.

Thus, the present study utilized an experimental procedure based on the Eriksen flanker task (Eriksen and Eriksen, 1974) that easily results in response errors and also provides feedback regarding the task response. Hence, this procedure allows for the dissociation of the neural mechanisms underlying the reconcentration process from those associated with error recognition. It was hypothesized that if participants could prepare an appropriate cognitive state by recognizing a previous error based on feedback from the previous trial, they would be able to appropriately respond to the subsequent task. In other words, an adjustment in terms of the concentration necessary for the task could be detected by whether the participant was able to successfully complete the trial immediately subsequent to a failed trial. In this manner, the cortical regions associated with the reconcentration of one’s own cognitive state could be differentiated from those necessary for observing negative feedback by observing the crucial instances in which the participant successfully completed the subsequent trial. Furthermore, the neural substrates necessary for reconcentration could be distinguished from the neural substrates required for the recognition of one’s own mistake, because the neural mechanisms that are activated during the latter situation would be associated with those commonly activated during the receipt of negative feedback.

Therefore, the present study utilized functional magnetic resonance imaging (fMRI) to explore the neural substrates involved in reconcentration to achieve an appropriate cognitive state. In addition, this study aimed to dissociate such neural substrates from those involved in the recognition of one’s own mistake.

## Materials and Methods

### Participants

The present study included 44 healthy Japanese volunteers (27 males and 17 females) with a mean age of 21.1 years (range: 19–25 years) that were judged to be right-handed according to the Edinburgh Handedness Inventory [18]. None of the participants had a history of neurological or psychiatric disease and all participants provided written informed consent prior to the experiment. The experimental protocol was approved by the Ethical Committee of the Tohoku University School of Medicine and the experiments were performed in compliance with national legislation and the Code of Ethical Principles for Medical Research Involving Human Subjects of the World Medical Association (Declaration of Helsinki).

### Experimental Task

**Figure 1:**
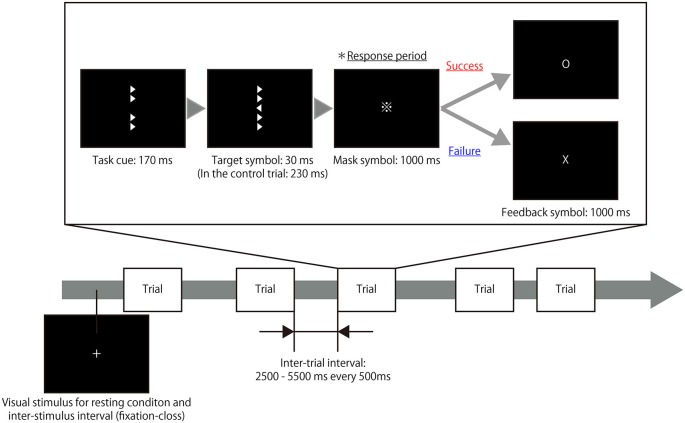
**Timeline of the experimental task.** A target symbol with congruent or incongruent flankers was presented after a task cue that consisted of only flanker symbols. The presentation time of the target symbol during the task trials (30 ms) differed from the presentation time during the control trials (230 ms). The participants were instructed to judge the direction of the target symbol and respond by pressing a button during the response period in which a mask symbol was presented. Following the response period, a feedback symbol indicated the result of the trial. Thus, the participant was aware of whether his/her response was correct or not in each trial. During the resting periods and inter-trial intervals, a fixation cross was presented at the center of screen.

The present study utilized a modified experimental procedure based on the Eriksen flanker task (Eriksen and Eriksen, 1974) and used right- and left-pointing triangles as target and flanker symbols. During each trial of the task, four flanker symbols were presented vertically for 170 ms in the areas surrounding the center of a computer screen as a task cue. Next, a target symbol with flanker symbols was presented in the center of the screen for an extremely short time (30 ms); immediately after the target symbol was shown, mask symbols were presented for 1000 ms to eliminate any afterimage effects. The right- and left-pointing triangles (target symbols) were presented in a random order. The participants were instructed to judge the direction of the target symbol and respond by pressing a button with either their right index finger or middle finger during the response period while the mask symbols were present. Subsequently, a feedback symbol that displayed the result of the trial was presented for 1000 ms and thus, the participant was aware of whether his/her response was correct or not for each trial. In control trials, the target symbol was presented for 230 ms so that the participants could easily determine the direction of the symbol, unlike in the task trial. The inter-trial intervals were set at 2500–5500 ms every 500 ms. During each inter-trial interval, the participants were asked to fix their gaze on a cross displayed in the center of screen. To ensure their motivation to complete the task, participants were also informed of the existence of a hidden score; that is, participants’ scores increased when they successfully completed a trial but decreased when they responded incorrectly on a trial. The rate of increase or decrease was magnified with repetitive successes or failures. Figure 1 illustrates the design of experimental task. To eliminate learning effects during the experimental task, the participants were asked to perform a training session outside of the fMRI room immediately after receiving the task instructions as well as a practice run during the actual fMRI scanning.

The fMRI experiment consisted of one short practice run and four experimental runs; each experimental run included 51 task trials and eight control trials. For each run, a 20 s rest period was imposed prior to the first trial and a 12 s rest period was imposed after the last trial. Thus, the total scanning time was 6 min and 22 s for each experimental run and 2 min and 4 s for each practice run. The participant was placed in a supine position in the MRI scanner and the stimulus presentation and response collection were performed using Presentation software (Neurobehavioral Systems; Albany, CA, USA). A semi-lucent screen was positioned behind the scanner and the visual stimuli were projected from outside the MRI room. The participant viewed the visual stimuli on the screen via a mirror attached to the head coil of the MRI scanner and a response pad was positioned at the participant’s waist so that the he/she could operate it comfortably with his/her right hand.

### fMRI Data Acquisition

All images were acquired using a Philips Achieva 3T MRI scanner (Philips; Amsterdam, Netherlands) and fMRI time series data covering the entire brain were acquired using T2*-weighted gradient-echo echo-planar imaging (GE-EPI). The parameters of the experiment were as follows: repetition *time*_(TR)_ = 2000 ms, echo *time*_(TE)_ = 30 ms, flip *angle*_(FA)_ = 80°, 32 slices, field of *view*_(FoV)_ = 192 × 192 mm, 64 × 64 matrix, slice *thickness* = 3 mm, and slice *gap* = 0 mm. For each participant, 251 scans were obtained during each experimental run and 62 scans were obtained during each practice run. To acquire a fine structural whole-head image, magnetization-prepared rapid-acquisition gradient-echo (MP-RAGE) images were obtained using the following parameters: *TR* = 6.5 ms, *TE* = 3 ms; *FA* = 8°; *FoV* = 240 × 240 mm, 240 × 240 matrix, 162 slices, and slice *thickness* = 1.0 mm.

### Exclusion Criteria Based on Behavioral Data

In order to investigate the neural mechanisms underlying participants’ recognition of their own response error and those necessary to reconcentrate their cognitive state when they recognized their error, two types of exclusion criteria were applied based on the behavioral data. Since the present study focused on the reconcentration process after recognizing an error, it is necessary to guarantee the number of the error trials included in each individual result as well as an appropriate behavioral accuracy. First, the data from six participants with low accuracy rates (<50%) throughout the experimental runs were discarded and second, the data from nine participants who performed too well (i.e., did not make at least two errors in a row during any of the runs) were also excluded. Additionally, the data from one participant was discarded due to a malfunction in response recording. Thus, the final analyses of the present study included the data of 28 participants (16 males and 12 females, mean age: 21.0 years, range: 19–24 years).

### fMRI Data Analysis

All preprocessing and statistical analyses of the fMRI data were carried out using statistical parametric mapping software (SPM8; Wellcome Trust Center for Neuroimaging, London, UK) implemented on Matlab (R2013b; Mathworks, MA, USA). The effects of head motion across the scans were corrected by realigning all images to the initial image and no data were excluded due to excessive head motion, defined as head motion >3 mm throughout each run. The lag due to scanning time for each slice was adjusted to the timing of the 16th slice and the structural image volume was then co-registered with the first EPI. All EPI were spatially normalized to the Montreal Neurological Institute (MNI)-T1 template using the parameter to co-register and normalize the structural image for the MNI-T1 template obtained by the segmentation process for each subject. Finally, each scan was smoothed with a Gaussian filter in a spatial domain with a full-width-at-half-maximum (FWHM) of 8 mm.

**Figure 2:**
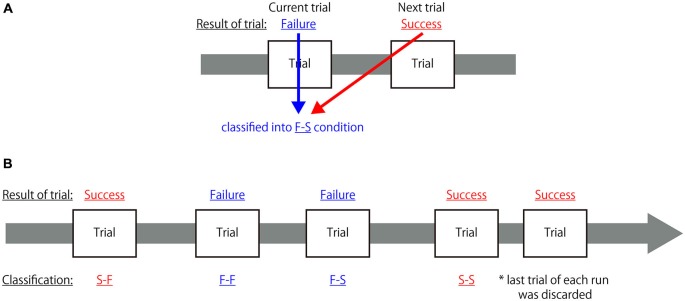
**Classification criteria for each trial in the first-level analysis. (A)** Sample explanation for the classification of a two-trial combination. If the participant failed to correctly respond to the current trial (Failure) but responded correctly to the subsequent trial (Success), that trial was classified as belonging to the F-S condition. **(B)** Example of classification of a combination of five trials. Each trial was classified into one of the four categories but, because the last trial of each run did not have a subsequent trial, it was not classified as belonging to any of these categories and was discarded from further analysis.

All fMRI data were analyzed using a two-level approach in SPM8. At the first level, the hemodynamic responses generated by a subject under the different experimental conditions were assessed at each voxel using a general linear model. In order to identify the neural mechanisms associated with reconcentrating to achieve an appropriate cognitive state when the participant recognized his/her own mistake, each task trial was grouped into four conditions based on the combination of successes and failures of the current and the subsequent trials; in other words, each trial was classified as: (1) a successful trial immediately followed by another successful trial (S-S); (2) a successful trial followed by failure on the next trial (S-F); (3) a failed trial followed by success on the next trial (F-S); and (4) a failed trial immediately followed by another failed trial (F-F). The classification criteria are depicted in Figure 2. It was hypothesized that cognitive activity indicating the preparation of an appropriate cognitive state for the next trial occurred during the receipt of feedback. Thus, the hemodynamic response was assumed to be the canonical hemodynamic response function and the onset of each hemodynamic response was set at the time when feedback was presented with a duration of 1 s from each onset. To eliminate an influence of task effects to perform congruent or incongruent flanker trials from the modeled response associated with feedback period, hemodynamic responses to the observation of each congruent and incongruent visual stimulus and to the button-press were also modeled as conditions of no-interest. The hemodynamic responses to the observation of each congruent and incongruent visual stimulus was assumed to be the canonical hemodynamic response function and the onset of each hemodynamic response was set at the time when the task cue was presented with a duration until the mask symbol was disappeared from the task cue, that is, 1.2 s from each onset for the regular trials and 1.4 s from each onset for the control trials. Similarly, the hemodynamic responses to the button-press was assumed to be the canonical hemodynamic response function and the onset of each hemodynamic response was set at the time when the participant pressed the button without duration. Additionally, the realignment parameters also included in the design matrix to exclude the effect of head motion. Global changes were adjusted by proportional scaling, and low-frequency confounding effects were removed using a high-pass filter with a 128 s cutoff. A multiple regression analysis was performed on each voxel to identify the regions where MR signal changes were correlated with the hypothesized model to obtain the partial regression coefficients of each voxel.

The second level of analysis was performed on an inter-subject basis using a two-way repeated-measures factorial analysis of variance (ANOVA) by using a flexible factorial design to configure a design matrix. One factor was the success or failure of the current trial and the other was the success or failure of the subsequent trial, and additionally the participant factor was also used. The contrast images obtained by the summation of the parameter estimate of each session were made every S-S, S-F, F-S and F-F conditions, and those images were used for this analysis. In order to elucidate specific activations that were associated with the preparation of an appropriate cognitive state following the recognition of one’s own mistake, a contrast image obtained by the subtraction of the (F-S > F-F) conditions was tested. Additionally, a contrast image obtained by the subtraction of the [(F-S + F-F) > (S-S + S-F)] conditions was also assessed to determine the difference in cortical activation between the occurrence of negative feedback vs. positive feedback. The statistical threshold for the second-level analysis was set at *p* < 0.05 and this was corrected for family-wise error (FWE) in the voxel level comparisons.

## Results

### Behavioral Findings

The average success rate for each trial was 0.682 when each attempt was considered to be independent. When the data were classified into the four described conditions based on the combination of successes and failures, the average probabilities of the S-S, S-F, F-S, and F-F trials were 0.482, 0.200, 0.202, and 0.116, respectively.

To confirm the presence of post-error adjustment effects, an average RT was calculated for each trial group based on the combination of the results of the present trial and the immediately preceding trial. Table 1A summarizes the average RT for each trial group. A 2 (the result of the trial immediately prior to the present trial) × 2 (the result of the present trial) repeated measures ANOVA revealed that the average RT for current failed trials was significantly slower than that of current successful trials (*F*_(1,27)_ = 11.176, *p* = 0.002) and that the average RT for current trials when the participant failed the previous trial was significantly slower than that of current trials when the participant successfully completed the previous trial (*F*_(1,27)_ = 4.738, *p* = 0.038). There was no significant interaction effect for the combination of the results of current and past trials (*F*_(1,27)_ = 0.509, *p* = 0.482).

**Table 1:** **The average reaction time (RT) for each trial group**.

(A) The average reaction time data [second] for the 2 × 2 repeated measures ANOVA (28 participants).					
		The result of the present trial			
		Success		Failed	
The result of the trial immediately prior to the present trial	Success	0.514		0.546	
	Failed	0.531		0.556	

**(B) The average reaction time data [second] for the 2 × 2 × 2 repeated measures ANOVA (27 participants)**.					
		**The result of the present trial for each trial type**			
		**Success**		**Failed**	
		**Congruent**	**Incongruent**	**Congruent**	**Incongruent**

The result of the trial immediately prior to the present trial	Success	0.502	0.527	0.571	0.534
	Failed	0.510	0.551	0.588	0.548

*Each trial was classified based on (A) the combination of the results of the present trial and the immediately preceding trial and (B) the combination of the results of the present trial for each congruent or incongruent trial type and the results of the immediately preceding trial*.

In addition, in order to examine an effect of congruency of the flanker task to the post-error adjustment effects, a 2 (the result of the trial immediately prior to the present trial) × 2 (the result of the present trial) × 2 (the congruence of the present trial) repeated measures ANOVA was also performed. An average RT was calculated for each trial group based on the combination of the results of the present trial for each congruent or incongruent trial type and the results of the immediately preceding trial. Since a missing value was found on data from one participant, data form twenty-seven participants were analyzed. Table 1B summarizes the average RT for each trial group. As a result, two main effects on factors associating with the result of the present trial (*F*_(1,26)_ = 16.330, *p* < 0.001) and the result of the immediately preceding trial (*F*_(1,26)_ = 4.794, *p* = 0.038) had significant difference. However, the remaining main effect of factor of congruence had no significant difference (*F*_(1,26)_ = 0.154, *p* = 0.698). Regarding each interaction effect, there was a significant interaction between the factors of the result of the present trial and congruence (*F*_(1,26)_ = 11.345, *p* = 0.001).

### fMRI Findings

**Figure 3:**
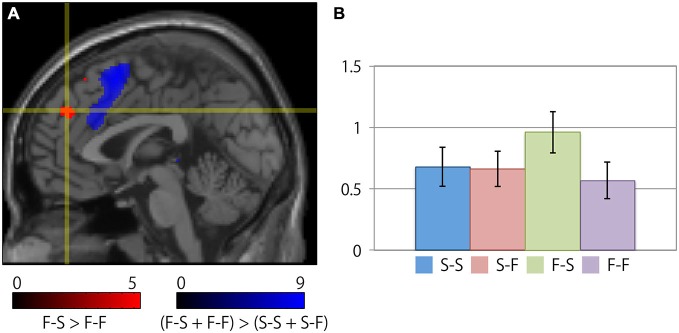
**Specific cortical activation during successful reconcentration for the next trial after recognition of negative feedback. (A)** The crosshair icon marks the location of the activation peak on a sagittal slice (*x* = 0 mm) on the MNI single subject template. The red and blue clusters represent the activation clusters obtained by the subtraction of the (F-S > F-F) conditions (red) and the [(F-S + F-F) > (S-S + S-F)] conditions (blue). The red and blue color scale indicates the *t*-values for the corresponding clusters. **(B)** Bar chart illustrating the percent signal changes within an activation cluster for each condition calculated using the MarsBar toolbox. The error bars represent the standard error of the mean.

Table 2 summarizes the cortical regions that showed significant activation following the subtraction of the (F-S > F-F) conditions. Specific activations were observed in the dorsal aspect of the MFC when the participant received negative feedback and gave a correct response on the next trial (F-S). Figure 3 shows the location of the activation peaks and the profiles of the local signal changes on the activation cluster. These trials exhibited a large increase in the blood oxygen level dependent (BOLD) signal relative to the other conditions.

**Table 2:** **Cortical areas showing significant activation when the subject received a failure feedback and gave a correct response in the next trial**.

Area	Brodomann’s area	Cluster size [voxel]	MNI coordinate [mm]			*t*-score
			*x*	*y*	*z*
Medial frontal gyrus	BA8	39	0	46	34	5.28
R. superior frontal gyrus	BA6	3	2	34	56	4.99
R. superior frontal gyrus	BA6	1	6	30	58	4.87

**Figure 4:**
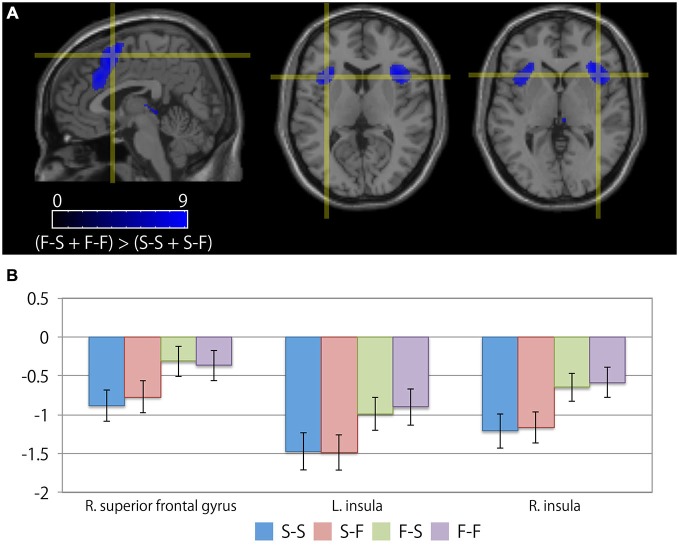
**Cortical regions exhibiting differences between the receipt of negative feedback indicating an error and the receipt of positive feedback. (A)** The crosshair icon marks the location of the activation peaks in the right superior frontal gyrus (left; sagittal slice: *x* = 4 mm), and the bilateral anterior insular regions (center; coronal slice: *y* = 16 mm, and right; coronal slice: *y* = 18 mm) on the MNI single subject template. The color scale indicates the *t*-values for the activation clusters. **(B)** Bar chart illustrating the percent signal changes within an activation cluster for each condition calculated using MarsBar toolbox. The error bars represent the standard error of the mean.

Table 3 summarizes the cortical areas that showed significant differences in activation following the subtraction of the [(F-S + F-F) > (S-S + S-F)] conditions. There was a significant increase in activity in the medial wall of the superior frontal regions extending to the anterior cingulate gyrus, the left lateral precentral gyrus, the right middle frontal gyrus, the intraparietal sulcus region, the bilateral insular cortices, and the medial part of the thalamus when the participants recognized their error based on feedback. In particular, the locations of the activation peaks in the MFC region were different from the activation peaks obtained by the subtraction of the (F-S > F-F) conditions even if a more liberal threshold (*p* < 0.001, uncorrected) was used. Figure 4 shows the location of each activation peak in the MFC and the insular regions as well as the profile of local signal changes for each activation cluster.

**Table 3:** **Cortical areas showing significant activation reflecting recognition of own mistake which was represented by comparison between conditions that failure feedback has been received vs. success feedback has been received in current trial**.

Area	Brodomann’s area	Cluster size [voxel]	MNI coordinate [mm]			*t*-score
			*x*	*y*	*z*
R. superior frontal gyrus	BA6	1593	4	12	56	9.14
R. anterior cingulate gyrus	BA32		8	26	32	8.93
R. insula	BA13	967	40	18	2	8.99
L. insula	BA13	664	−34	16	8	8.08
R. middle frontal gyrus	BA9	8	46	16	28	5.02
L. precentral gyrus	BA6	148	−44	2	32	6.35
L interior parietal lobule	BA40	22	−32	−52	44	5.34
		5	−44	−34	44	4.97
R. thalamus		37	4	−30	0	5.19
L. thalamus			−4	−30	−2	5.21
		1	−12	−20	10	4.92

In order to depict a relationship between a cortical activation reflecting successful reconcentration process and behavioral change, a correlation analysis between the local activation profiles of the F-S condition on the activation cluster of the dorsal aspect of the MFC and the average RT for successful trials following failed trials was examined. As a result, although a weak negative correlation (*R* = −0.304) was observed, there was no statistically significant correlation (*p* = 0.116).

## Discussion

The present findings demonstrated a significant increase in activation in the dorsal aspect of the MFC when participants received feedback regarding a failed trial and then responded correctly on the subsequent trial. However, this specific increase in activation was not observed when participants succeeded in adjacent trials. Thus, the dorsal aspect of the MFC plays an important role during reconcentration to prepare an appropriate cognitive state for the next trial subsequent to an error. In contrast, cortical activation that reflected the recognition of one’s own behavioral error, as represented by the comparison between trials in which negative feedback was received vs. trials with positive feedback, was primarily observed in the posterior aspect of the MFC and the bilateral insular regions. Additionally, the location of this MFC activation differed from that of the activation focus that was observed when participants received negative feedback and then responded correctly on the next trial. Thus, the present findings indicate that the neural substrates associated with the preparation of an appropriate cognitive state following the receipt of feedback regarding one’s own error differ from those involved in the recognition of one’s own behavioral error, and that these substrates exist separately in the MFC. Even if the participant recognized his/her own error, the next error would not be prevented if the neural substrates necessary to prepare the appropriate cognitive state could not be engaged. Since the present finding indicated distinct cortical activation different from activation which was involved in trials subsequent to error trial suggested by previous studies, it could contribute to understand cognitive control to fulfill a successful post-error adaptation.

The present study used fMRI data to model hemodynamic responses during the receipt of feedback representing the result of a particular trial. The parameter estimates for the S-S, S-F, F-S, and F-F conditions were considered to depict changes in cognitive processing dependent upon participants’ awareness of their own performance based on feedback information. Additionally, the hemodynamic responses associating with the observation of each kind of visual stimulus and the button-press were also modeled to eliminate the task effect of congruent or incongruent trials. Because both the F-S and F-F conditions included a failed trial and the receipt of negative feedback, the difference between the F-S and F-F conditions consisted of whether the participant responded correctly in the trial immediately following the failed one. The behavioral results revealed that there was a post-error slowing of the RTs in the trials subsequent to the failure trials and therefore, the cognitive mechanisms representing post-error adjustments were engaged during the trial immediately following a failed trial. Taken together, the dorsal aspect of the MFC became active only when participants successfully prepare the cognitive state to respond correctly on the next trial, it did not participate in only the process which makes the post-error slowing.

Because the present study focused on the cognitive processing that occurred when a participant recognized his/her own mistake based on feedback, the observed cortical activity should have occurred in advance of the cognitive processing that reflected post-error adjustment. Several fMRI studies have investigated the neural substrates involved in post-error adjustment by examining changes in cortical activity during the post-error period (Hester et al., 2007; Li et al., 2008; Danielmeier et al., 2011). Furthermore, event-related potentials (ERPs) studies demonstrated the relationship between occurrence of post-error slowing and larger N2 activity, and this activity was not related to a cognitive processing of error-monitoring (Soshi et al., 2014) or post-conflict slowing (Chang et al., 2014). Based on the model describing the dual-networks architecture of top-down control (Dosenbach et al., 2008), the top-down adjustment, or initiation of cognitive control, is represented as activation within the lateral parietofrontal network while the set maintenance for goal-directed behavior is associated with the cingulo-opercular network. In this manner, the cognitive processing involved in reconcentration could be considered a regulatory activity that precedes top-down control in order to prevent future mistakes because reconcentration should influence post-error adjustment. Accordingly, in the present study, the activation associated with reconcentration was observed during the feedback period of the preceding trial. Thus, the present findings imply that the dorsal aspect of the MFC plays an important role in the meta-control process by adjusting the top-down attentional control that is introspectively induced by the recognition of one’s own error. In the present study, the subtraction of the (F-S > F-F) conditions was considered to have represented cortical activation associated with successful reconcentration while preparing post-error adjustment after recognizing one’s own mistake. Therefore, this mechanism acts as a trigger to prepare successful post-error behavioral adjustments.

Furthermore, the error-related areas of activation obtained by the subtraction of the [(F-S + F-F) > (S-S + S-F)] conditions did not reveal any specific signal changes during the F-S condition based on the results of a region-of-interest (ROI) analysis. With respect to the MFC, significant levels of activation were observed in the more posterior portion of this region and this activation did not overlap with the significant areas of activation observed during the F-S condition. Thus, the present results demonstrated that the neural substrates underlying the reconcentration process when preparing an appropriate cognitive state can be dissociated from the neural substrates associated with error-related processing. In the F-S condition, the participants internally modulated their cognitive state for the next trial after receiving negative feedback regarding their own behavior. Thus, a self-induced re-adjustment of one’s own cognitive state during the experimental task was expected to occur. Gusnard et al. (2001) observed increased activation in the anterior aspect of the dorsomedial prefrontal cortex (dmPFC) induced by an inner-cued choice task, and concluded that this activation reflected the presence of self-referential mental activity related to the execution of the experimental task. Additionally, a meta-analysis of neuroimaging studies investigating self-referential processes indicated that the dorsal aspect of the MFC was related to reappraisal and the evaluation of self-related stimuli (Northoff et al., 2006). Taken together, these and the present findings suggest that the activation of the dorsal aspect of the MFC represents an introspective process that confirms or readjusts one’s own cognitive state to allow for the preparation of an appropriate cognitive state to successfully complete a task. From the result of the correlation analysis, we have not obtained significant correlation between the local activation profiles on the MFC cluster reflecting reconcentration process and the behavioral changes corresponding to the reconcentration process, even though the weak negative correlation was observed. Li et al. (2008) reported that several activation foci on right prefrontal cortex involved in post-error slowing in RT. King et al. (2010) also reported the post-error adjustment process such as post-error slowing and post-error reduction of interference were separately mediated activation of distinct cortical regions. This difference between the present result and previous findings might be caused by the experimental procedure, especially, in the participants’ exclusion criteria. The present study excluded the data from participants with low accuracy late and with participants who performed too well to ensure the number of trials for investigating each condition. Thus, variance of data might be slightly insufficient compared with individual differences to analyze the correlation. Further investigation will be needed to elucidate the relation of the magnitude of activity reflecting the reconcentration process to behavioral changes as a result of the reconcentration.

In previous neuroimaging studies, the flanker task has been widely utilized to investigate the neural substrates associated with the recognition of incongruent stimuli or response competition (Botvinick et al., 1999, 2004; Casey et al., 2000; Ullsperger and von Cramon, 2001; van Veen et al., 2001; Debener et al., 2005). This task has also been used to clarify the neural mechanisms underlying response inhibition (Bunge et al., 2002; Wager et al., 2005; Blasi et al., 2006; McNab et al., 2008). These findings indicate that the dorsal anterior cingulate and the medial prefrontal cortices participate in such behaviors. However, these studies primarily focused on the cortical activation that occurs when congruent or incongruent flanker stimuli are presented and on the differences between these two conditions and as a result, the construction of the hypothesized hemodynamic response models differed from that of the present study. With regard to the activation focus being localized in the MFC, the activation peak obtained by the subtraction of the (F-S > F-F) conditions was observed in a more anterior portion of the MFC relative to other studies using the flanker task. From the result of ANOVA for the behavioral measures, the main effect on factor associating with the result of the immediately preceding trial was significant even if the factor of task congruence was considered. Although the significant interaction effect between the factors of the result of the present trial and congruence was observed, there was no tendency that the RT becomes shorter when the participant failed the trial from our behavioral data. Thus, it is indicated that a post-error slowing effect was robustly observed in the experiment despite the result of subsequent trial. In contrast to these previous findings, the cortical activation obtained by the subtraction of the (F-S > F-F) conditions likely reflected the neural substrates underlying the successful reconcentration process when negative feedback was received.

In the present study, significant activation associated with the recognition of one’s own mistake was observed in the posterior aspect of the MFC extending into the anterior cingulate gyrus and the bilateral anterior insular cortices. This activation cluster in the MFC was located on a more posterior side relative to the activation cluster that was observed when participants successfully reconcentrated during the experimental task. Because the present experimental task provided visual feedback regarding the results of each trial, the neural substrates necessary for perceiving negative feedback were revealed by comparing trials involving negative feedback with those involving positive feedback. Previous reviews of neuroimaging studies have suggested that the cognitive function of the posterior portions of the MFC regions that extend to the anterior cingulate cortex are associated with cognitive control during the processing of negative feedback (Ridderinkhof et al., 2004) as well as with conflict monitoring (Botvinick et al., 2004). Additionally, the bilateral anterior insular regions are considered to be a part of the attentional network (Dosenbach et al., 2008), and changes in cortical activity in these regions have a strong relationship with cognitive control during error processing (Menon et al., 2001; Debener et al., 2005; Ullsperger et al., 2010; Klein et al., 2013). Thus, the present findings are consistent with those of previous studies in that the activation of the posterior portions of the MFC and the anterior insular cortices reflect error processing during the receipt of failure feedback.

Furthermore, Neta et al. (2014) reported that the left dorsolateral prefrontal cortex, the posterior parietal region, and the thalamus also exhibit significant activation associated with error processing. These authors have suggested that parietofrontal activation is involved in task control and that the occurrence of error processing modulates the magnitude of that activation, Ide and Li (2011) reported that medial part of thalamus has functional connectivity with ventrolateral prefrontal cortex, supplementary motor area and cerebellum, and this connectivity mediates error and post-error processing. The present findings support this assertion, because the activation of the parietofrontal network and medial part of thalamus was enhanced by the occurrence of error recognition when negative feedback was presented and because this activation did not influence the result of the subsequent trial.

In conclusion, the present study demonstrated that activation in the dorsal aspect of the MFC was associated with the reconcentration process necessary for preparing an appropriate cognitive state to prevent future errors. Moreover, this neural substrate was clearly dissociated from the neural substrates in other regions of the MFC that were involved in error recognition. Thus, humans can prevent the performance of successive errors if the dorsal aspect of the MFC is appropriately engaged when recognizing their own mistake. With respect to human factors research, human errors may be minimized by designing a human-machine system that can appropriately engage this neural substrate.

## Funding

This study was supported by JSPS KAKENHI Grant Number 24700121.

## Conflict of Interest Statement

The authors declare that the research was conducted in the absence of any commercial or financial relationships that could be construed as a potential conflict of interest.
